# ARDS severity in COVID-19: a case–control study of laboratory biomarkers and IL-10 SNP analysis

**DOI:** 10.48101/ujms.v130.11515

**Published:** 2025-07-07

**Authors:** Shukur Wasman Smail, Niaz Albarzinji, Karim Jalal Karim, Rebaz Hamza Salih, Christer Janson

**Affiliations:** a Department of Biology, College of Science, Salahaddin University-Erbil, Erbil, Iraq; b College of Pharmacy, Cihan University-Erbil, Erbil, Iraq; c College of Medicine, Hawler Medical University, Erbil, Iraq; d Department of Medical Laboratory Science, Faculty of Science and Health, Koya University, Erbil, Iraq; e Department of Respiratory Medicine, PAR Private Hospital, Erbil, Iraq; f Department of Medical Science, Respiratory Medicine, and Allergology, Uppsala University and University Hospital, Uppsala, Sweden

**Keywords:** Acute respiratory distress syndrome, COVID-19, cytokines, hematological parameters, polymorphism

## Abstract

**Background:**

Acute respiratory distress syndrome (ARDS), which is often observed in severe cases of coronavirus disease 2019 (COVID-19), is known to be a major contributor to higher mortality rates. This study assesses how hematological parameters, inflammatory biomarkers, cytokines, and the –1,082 A/G polymorphism are associated with ARDS severity in COVID-19 patients.

**Methods:**

Following exclusions, a 6-month prospective case–control study included 82 healthy controls (HCs) and 158 COVID-19 patients with varying severities of ARDS (mild: 73, moderate: 53, and severe: 32). Blood samples were collected at admission, and laboratory biomarkers were assessed using various methods. Statistical analyses included one-way analysis of variance with Tukey’s test for group comparisons, Pearson correlation, and receiver operating characteristic curve for analyzing independent associations with COVID-19 severity. Multiple linear regression and chi-square tests were used to evaluate quantitative outcomes and categorical associations, respectively.

**Results:**

Severe ARDS patients exhibited higher C-reactive protein (CRP) levels compared to HCs. Compared to HCs, patients with moderate and severe ARDS had higher neutrophil to lymphocyte ratio (NLR), neutrophil counts, tumor necrosis factor-alpha, and interleukin-10 (IL-10), as well as lower lymphocyte counts and reduced partial pressure of oxygen/fraction of inspired oxygen (PaO_2_/FiO_2_) ratio. IL-10, body mass index, CRP, and NLR were associated with reduced PaO_2_/FiO_2_ ratio. IL-10 and CRP had the highest area under curve values toward ARDS severity. COVID-19 patients possessing the –1,082 A/G single nucleotide IL-10 GG and GA genotypes and the G allele presented with less severe ARDS.

**Conclusion:**

Hematological indices (neutrophil count and NLR), CRP, and serum IL-10 hold promise in monitoring ARDS severity in COVID-19 patients. In addition, COVID-19 patients with GG and AG genotypes and the G allele of the IL-10 gene’s–1,082 A/G polymorphism experience less severe ARDS. This highlights the potential protective role of IL-10 genetic variation in modulating the severity of inflammatory responses during severe acute respiratory syndrome-coronavirus-2 infection and may serve as a useful genetic marker for risk stratification in clinical settings.

## Introduction

The coronavirus disease 2019 (COVID-19) has risen as a pivotal global health challenge since its initial detection in Wuhan, city of China, within the final month of 2019, which is regarded as a lethal disease. The etiological agent for COVID-19 is severe acute respiratory syndrome-coronavirus-2 (SARS-CoV-2), which is characterized by an enveloped structure housing a positive-sense, single-stranded Ribonucleic Acid (RNA). The coronavirus family has the potential to mutate and spread among vulnerable populations, thus presenting a worldwide risk (1). At the last update of the Coronavirus Tracker on April 13, 2024, 229 countries and territories have reported 704,753,890 confirmed COVID-19 cases and 7,010,681 deaths (2).

Acute respiratory distress syndrome (ARDS) not only is typically associated with sepsis caused by bacterial infections but can also stem from viral infections like SARS-CoV-2. The development of ARDS in the context of SARS-CoV-2 is thought to occur through either the virus’s direct invasions on respiratory epithelial cells or through an amplified immune response (3) resulting in the overproduction of cytokines such as interleukin-10 (IL-10) and tumor necrosis factor-alpha (TNF-α), as well as increased levels of neutrophils, C-reactive protein (CRP), and neutrophil to lymphocyte ratio (NLR), while the lymphocyte count decreases (4–7). Hence, the assessment of laboratory biomarkers in COVID-19 patients with ARDS becomes crucial for predicting disease severity, facilitating early intervention, and preventing disease progression.

When the severity of the infection and pulmonary compromise reach a point where gaseous exchange is no longer feasible, the patient may necessitate invasive mechanical ventilation and/or extracorporeal membrane oxygenation for life support (8). The NLR and concentrations of CRP are indicative measures employed in gauging the potential intensity and prognosticating of certain viral diseases (9). The research indicated that the initial median NLR was markedly elevated in patients with COVID-19 who subsequently progressed to ARDS, as opposed to patients who did not experience ARDS development (6). Smilowitz, Kunichoff (10) reported that elevated levels of CRP concentrations exceeding the median value have been correlated with an increased likelihood of critical illness and mortality risk, in contrast to cases where CRP levels fall below the median threshold.

ARDS is heavily influenced by the action of TNF-α, a cytokine implicated in initiating inflammatory responses. The presence of TNF-α leads to a cascade of inflammatory mediators, potentially resulting in cytokine storm, and subsequent inflammation within the lung tissue (11). TNF-α not only impairs the structural integrity of pulmonary blood vessels, resulting in the development of pulmonary edema (12) but also induces thrombogenesis, leading to obstructions in the lung’s microvasculature (13). IL-10, a proinflammatory cytokine, expressed greatly in the early phase of ARDS. IL-10 facilitates the development of ARDS by preventing the differentiation of bone marrow-derived stem cells into alveolar type 2 (AT II) epithelial cells (5). IL-10 plays a critical role in pulmonary disorders by triggering inflammatory cascades and nitric oxide synthase. The buildup of nitric oxide exacerbates the severity of ARDS (14, 15).

A single nucleotide polymorphism (SNP) in the promoter region of the IL-10 gene at position –1,082, where an adenine is replaced by guanine (A to G), plays a significant role in controlling IL-10. Those homozygous for the G allele (–1,082 GG) exhibit increased levels of circulating IL-10 (16, 17). Individuals possessing the GG genotype might exhibit a heightened likelihood of ARDS manifestation, with the risk potentially varying according to age. Individuals under the age of 52 carrying the GG genotype have a higher likelihood of developing ARDS. Conversely, in examined patients aged 52 years and older, the –1,082 GG genotype was shown to offer a protective effect against ARDS (18). Various pharmacological treatments have undergone clinical trial assessments to manage the clinical manifestations of ARDS, yet their efficacy remains unconfirmed. The knowledge gap lies in the absence of reliable prognostic markers for predicting which COVID-19 patients are likely to develop ARDS, particularly from a genetic perspective. While current research has focused on clinical and inflammatory markers, the role of genetic polymorphisms, such as the –1,082 A/G polymorphism of IL-10, remains underexplored. Our study bridges this gap by investigating how integrating hematological parameters (neutrophil, lymphocyte, and NLR), cytokine profiles (IL-10 and TNF-α), and genetic data (1,082 A/G polymorphism of IL-10) can provide early prognostic insights. Identifying patients predisposed to severe ARDS allows for targeted monitoring and timely intervention, potentially preventing progression to critical stages.

## Materials and methods

### Research design

**Figure 1:**
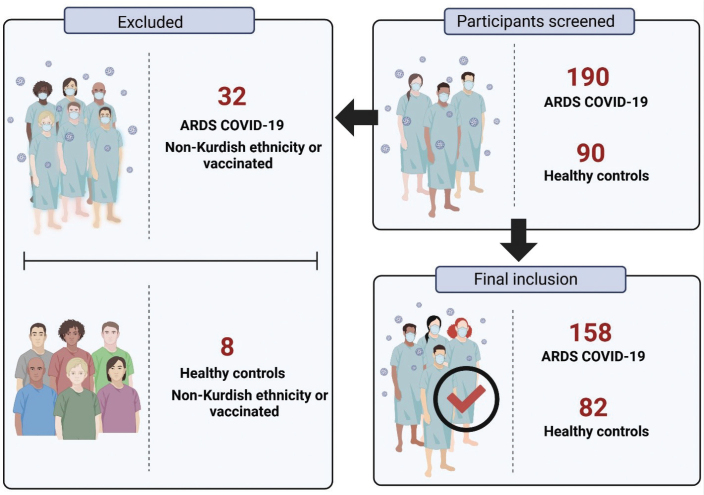
Study participant selection workflow. The diagram illustrates the selection process for the study participants. A total of 190 ARDS COVID-19 patients and 90 healthy controls were initially screened. After excluding 32 ARDS patients and 8 healthy controls due to non-Kurdish ethnicity or vaccination status, the final study included 158 ARDS COVID-19 patients and 82 healthy controls. ARDS: Acute respiratory distress syndrome.

This prospective case–control study was conducted over 6 months, from June 3 to November 3, 2021, and a total of 190 ARDS COVID-19 patients and 90 healthy controls (HCs) were initially screened. After excluding 32 ARDS patients and 8 HCs due to non-Kurdish ethnicity or vaccination status, the final study included 158 ARDS COVID-19 patients and 82 HCs (Figure 1).

The inclusion criteria were the COVID-19 patients with ARDS (86 males/72 females) with a mean age of 43.51 ± 10.83, who were tested positive for SARS-CoV-2 and visited the Emergency Departments of hospitals (Lalav hospital-Erbil, Shahid Hemn hospital-Sulaymaniyah, Burn hospital-Duhok) in the Kurdistan Region-Iraq (Table 1). In that timeframe, the alpha variant (VOC/B.1.1.7) of SARS-CoV-2 predominated (19). HCs (*n* = 82, 39 males/43 females) with a mean age of 40.78 ± 12.53 were enrolled in the current study. The HC group had a mean age of 40.78 ± 12.53 younger than the ARDS COVID-19 group (43.51 ± 10.83), but this difference was not statistically significant (*P* = 0.08). A significant difference in age was only observed between those with severe ARDS COVID-19 and HCs (Table 1).

**Table 1:** A comparison of demographic factors and laboratory biomarkers among HCs and various ARDS groups of COVID-19 patients.

Parameters	HCs Mean ± SD	Mild ARDS COVID-19 Mean ± SD	Moderate ARDS COVID-19 Mean ± SD	Severe ARDS COVID-19 Mean ± SD	*P*
*N*	82	73	53	32	-
Gender(M/F)	39/43	40/33	30/23	16/16	0.341
Age (Years)	40.78 ± 12.53	39.42 ± 11.05	43.69 ± 9.32	52.53 ± 6.41	<0.001
BMI (Kg/m^2^)	26.78 ± 4.62	26.59 ± 4.39	30.41 ± 6.33	29.52 ± 5.67	<0.001
TNF-α (pg/ml)	6.402 ± 2.68	12.61 ± 8.29	9.947 ± 6.57	20.45 ± 7.44	<0.001
IL-10 (pg/ml)	1.444 ± 1.2	2.359 ± 1.54	6.567 ± 2.1	10.74 ± 4.85	<0.001
CRP (mg/l)	0.776 ± 0.87	5.613 ± 14.39	27.68 ± 56.55	68.51 ± 32.47	<0.001
Lymphocyte (10^3^/µL)	2.095 ± 0.59	1.323 ± 0.53	1.461 ± 0.45	1.012 ± 0.61	<0.001
Neutrophil (10^3^/µL)	4.252 ± 1.11	8.467 ± 3.96	6.763 ± 2.62	14.68 ± 6.12	<0.001
NLR	2.130 ± 0.71	7.617 ± 5.06	5.381 ± 3.76	19.00 ± 11.17	<0.001
PaO_2_/FiO_2_	405.4 ± 3.86	268.5 ± 27.06	151.6 ± 30.66	71.31 ± 17.55	<0.001
Co-morbidities					
Multiple morbidities	-	10	13	7	-
COPD	-	-	1	1	-
DM	-	3	2	4	-
HTN	-	8	5	7	-
OB	-	11	14	5	-
RA	-	2	1	-	-
ESRD	-	-	1	1	-

Comparison of variables among groups was done by one-way ANOVA, and post-comparison was done by Tukey test. A statistical significance was assumed if the *P*-value was less than 0.05. BMI: body mass index; CRP: C-reactive protein; HC: healthy control; IL-10: interleukin-10; TNF-α: tumor necrosis factor-alpha; NLR: neutrophil to lymphocyte ratio; PaO_2_/FiO_2_: partial pressure of oxygen/fraction of inspired oxygen; ARDS: acute respiratory distress syndrome; COVID-19: coronavirus disease 2019; M: male; F: female; N: sample size; COPD: chronic obstructive pulmonary disease; DM: diabetes mellitus; HTN: hypertension; OB: obesity; ESRD: end stage renal disease; SEM: standard error of mean; SD: standard deviation; ANOVA: analysis of variance; RA: Rheumatoid Arthritis.

COVID-19 patients were randomly chosen and confirmed using reverse transcriptase-polymerase chain reaction (RT-PCR) and radiological imaging (20). They were subsequently categorized into three distinct groups according to the degree of hypoxemia (reduced partial pressure of oxygen/fraction of inspired oxygen (PaO_2_/FiO_2_)): mild ARDS COVID-19 (*n* = 73, 40 males/33 females), moderate ARDS COVID-19 (*n* = 53, 30 males/23 females), and severe ARDS COVID-19 (*n* = 32, 16 males/16 females) (21) and updated in 2023 (22). In the current study, mild ARDS was characterized by a PaO_2_/FiO_2_ ratio ≤ 300 and >200 mm Hg. Moderate cases of ARDS were characterized by a PaO_2_/FiO_2_ ratio ≤ 200 and >100 mm Hg. Severe cases of ARDS were defined by a PaO_2_/FiO_2_ ratio ≤ 100 mm Hg. For the purpose of predicting severity of disease based on –1,082 G/A genotype of IL-10, the COVID-19 study participants were re-categorized into two non-severe ARDS COVID-19 groups (mild and moderate ARDS COVID-19, *n* = 126) and those who have severe ARDS COVID-19 (*n* = 32).

The hospital’s record system supplied information on the patient population characteristics, their medical histories, and associated health disorders. Research protocols were accepted by the ethical committee (No. 01062021-5-9) at the University of Duhok and Ministry of Health/Directorate General of Health-Duhok. It is consistent with the Declaration of Helsinki. Each participant was presented with a consent form, and if the patients were unable to sign due to the severity of their illness or disability, their legal representatives were asked to affix their signature.

### Inclusion criteria for participants

During the study period (June 3 to November 3, 2021), the vaccination rollout in the region was in its early stages, and only a small proportion of the population had been vaccinated. To minimize potential confounding factors, we chose to exclude vaccinated individuals from the study.

In this study, both the ARDS group and HCs were of Kurdish ethnicity. Since the Kurdistan region of Iraq includes diverse ethnicities (Kurdish, Arab, Christian, and Turkmen), each with different genetic backgrounds, we selected only Kurdish participants as they constitute the majority of the population, thereby decreasing the risk of bias and ensuring more reliable results.

Inclusion criteria

During this research, enrollment was limited to ARDS COVID-19 patients who fulfilled the following inclusion criteria:

1. They were unvaccinated, and they experienced ARDS.
2. Their RT-PCR tests came back positive for SARS-CoV-2.
3. They had complete demographic data and samples (blood and nasopharyngeal).
4. They have accepted and signed a consent form.
5. The entire group shared a common ethnicity, which was Kurdish.

Exclusion criteria

Patients were excluded from the study if:

1. They had been immunized against COVID-19 or had a PaO_2_/FiO_2_ ratio exceeding 300.
2. Had negative RT-PCR test results for SARS-CoV-2.
3. Lacked complete demographic data or required samples (blood and nasopharyngeal).
4. Did not provide consent or declined to sign the consent form.
5. Where not of Kurdish ethnicity.

Inclusion criteria for healthy controls

HCs without COVID-19 symptoms were recruited from the hospital. We conducted RT-PCR tests to verify that they were negative for COVID-19 and were not infected with SARS-CoV-2. We used this selection process to create a reliable control group to compare against COVID-19 patients. To avoid confounding research results, HCs are selected based on the absence of comorbidities like diabetes, hypertension, or cardiovascular disease, as these conditions can affect immune responses and other physiological parameters (23).

The study’s HCs met these criteria:

1. The RT-PCR test result for SARS-CoV-2 from the nasopharyngeal swab returned a negative result.
2. No clinical signs indicative of a pulmonary infection.
3. No previous records of chronic illnesses.
4. Non-smokers.
5. Normal hematological counts.
6. Normal levels of CRP and D-dimer.

### Sample and data collection

All participants provided a blood sample upon their arrival, prior to receiving any medication. The phlebotomy was completed using a 10-milliliter syringe to collect venous blood. This blood was then transferred into serum-separating tubes (SSTs) as well as into ethylenediaminetetraacetic acid (EDTA) tubes. Subsequent to the centrifugation of SST tubes at 3,000 rpm for a duration of 15 min for serum separation, respective aliquots were transferred into Eppendorf tubes and stored at –80°C in preparation for later examinations. The blood contained in the EDTA tube was promptly used to perform a complete blood count and conduct genetic testing.

### Data collection and parameter determination

Demographic information, including gender, age, and body mass index (BMI), in addition to comorbidities, was obtained from the system database of the hospital. The BMI was determined by dividing the individual’s weight in kilograms by their height in meters squared. Hematological analyzer, named Sysmex XT-2000i (Japan), was utilized to assess counts of hematological parameters (neutrophil and lymphocyte). NLR was calculated by dividing the neutrophil counts by the lymphocyte counts. CRP was evaluated by Cobas c311 (Roche Diagnostics, Switzerland). The evaluation of CRP is based on immunoturbimetric assay (24). The PaO_2_/FiO_2_ was calculated by special emergency physicians in the intensive care unit (ICU) unit based on mathematical equation (25). The sandwich enzyme-linked immunosorbent assay (ELISA) technique was used for measuring IL-10 (Elabscience, USA) and TNF-α (MyBiosoure, USA) that were used throughout this study.

### DNA extraction and genotyping of –1,082 G/A SNP of IL-10

The extraction of deoxyribonucleic acid (DNA) was carried out following the guidelines provided by the specified manufacturer (Jena Bioscience, Germany). DNA sample purity and concentration were assessed with NanoDrop spectrophotometer (Thermo Fisher Scientific, Waltham, United States), in ng/µl unit. The range of concentration and purity of DNA were 13.39–125.68 ng/µl and (1.62–1.93), respectively. The genotyping of –1,082 G/A SNP of IL-10 was done by allele-specific (AS)-PCR, and the primers were obtained from other studies (26, 27) and checked by NCBI primer blast (Supplementary Table 1). The thermal cycle condition and procedure of Allele-Specific-Polymerase Chain Reaction (AS-PCR) were done according to the study conducted by Qadir, Pirdawid (27). The PCR-generated product was then subjected to gel electrophoresis by utilizing a 2% gel, subsequently stained with ethidium bromide, and with the aid of UV light for visualization in a gel doc system. Each sample was tested with two reaction tubes. If both tubes produced a band of 258 bp, it indicates that the sample has an AG genotype. Furthermore, in cases where the 258 band is only present in the G allele reaction tube, the sample indicates a GG genotype. Conversely, if the 258 band is only observed in the A allele reaction tube, the sample indicates an AA genotype (Supplementary Figure 1).

### Statistical analysis

Normality tests, including the Shapiro-Wilk, Kolmogorov-Smirnov, and De-Agostino tests, were conducted using GraphPad Prism version 9 (GraphPad Software, USA) to assess whether the data followed a parametric distribution or not. As data passed normality tests, we applied all parametric tests for them. Data were displayed as mean (M) ± standard deviation (SD). To compare groups, a one-way analysis of variance (ANOVA) was used. For multiple comparisons following ANOVA, the Tukey test was employed as a post-hoc analysis. The Pearson correlation was used to assess the correlation between variables. The receiver operating characteristic (ROC) curve was utilized to evaluate which parameters could predict the severity of ARDS in COVID-19 patients. A ROC curve in MedCalc 20 (MedCalc Software, Belgium) provided values for area under curve (AUC), cut-off, specificity, sensitivity, positive predictive value (PPV), and negative predictive value (NPV). When the dependent variable was quantitative, multiple linear regression was utilized in SPSS 29 (IBM, USA). Standardized beta (β) coefficients are regression coefficients obtained after standardizing all variables (independent and dependent) to have a mean of 0 and a SD of 1 (28, 29).

To investigate the association between *IL-10 –1,082 A/G* polymorphism and the severity of ARDS in COVID-19 patients, both unadjusted and adjusted statistical methods were applied. Unadjusted odds ratios (ORs), 95% confidence intervals (CIs), and *P*-values were calculated using the chi-square test to assess the crude associations between genotypes and ARDS severity. For adjusted analyses, multivariable logistic regression models were constructed to estimate adjusted odds ratios (aORs) and corresponding 95% CIs, controlling for potential confounding factors including age, gender, and BMI. B and standard errors of the mean (SEM) were also reported. Genotype AA served as the reference group for all genotype comparisons. Genetic models included individual genotype comparisons (AG vs AA and GG vs AA), a dominant model (AG+GG vs AA), a recessive model (GG vs AA+AG), and an allele model (G vs A). Adjusted ORs were calculated using the formula aOR = e^B^ (where e is the mathematical constant approximately equal to 2.71828), and 95% CIs were computed as e^B±1.96×SEM^ (30). A statistical significance was assumed if the *P*-value was less than 0.05.

## Results

### Participants and research specimens

In this study, a group of 240 individuals was examined, of which 158 participants (65.83%) had COVID-19 along with ARDS, while the remaining 82 subjects (34.17%) were HCs. Among those who tested positive, 73 individuals (46.20%) exhibited mild ARDS symptoms of COVID-19, 53 subjects (33.54%) presented with a moderate level of ARDS COVID-19, and there were 32 cases (20.26%) of severe ARDS COVID-19 as classified by the clinical criteria of the Berlin definition (21).

Table 1 represents the demographical characteristics and comorbidities of patients with ARDS from COVID-19, alongside a comparison with HCs. The average age of HCs was 40.78 ± 12.53 years; there were 39 men (47.56%) and 43 women (52.44%). Among ARDS COVID-19 positive patients, the average age was 43.51 ± 10.83 years; this group comprised 86 men (54.43%) and 72 women (45.57%).

Non-significant differences in CRP levels were observed between the HC and mild ARDS groups (*P* = 0.256). Significant differences (*P* < 0.0001) were found between HCs and the moderate and severe ARDS COVID-19 groups. All groups with ARDS related to COVID-19 showed a marked rise in the NLR and neutrophil counts having *P*-value < 0.0001, and this was coupled with a pronounced reduction in lymphocyte counts when compared with the HCs, as presented in Table 1 and Supplementary Table 2.

### Serum levels of IL-10 and TNF-α in patients afflicted with ARDS due to COVID-19 infection

When comparing the serum concentrations of TNF-α and IL-10 between HCs and patients, it was found that patients with moderate and severe ARDS caused by COVID-19 had significantly higher levels of IL-10 (*P* < 0.001). The IL-10 levels were recorded as 6.567 ± 2.1 for moderate cases and 10.74 ± 4.85 for severe cases, whereas HCs had levels at 1.444 ± 1.2. Conversely, no significant difference was noted in IL-10 levels when comparing the mild group (2.359 ± 1.54) with HCs (1.444 ± 1.2) (*P* = 0.066). Corresponding findings were seen with TNF-α. In all ARDS groups of COVID-19 patients, levels of TNF-α were markedly elevated (*P* < 0.001) as compared to the HCs, as displayed in Table 1 and Supplementary Table 2.

### The interplay of laboratory biomarkers shows a significant correlation with PaO_2_/FiO_2_.

To gain a comprehensive understanding of the demographic factors and laboratory biomarkers’ impact on ARDS in COVID-19, we conducted a correlation analysis between age, BMI, CRP, hematological biomarkers (neutrophil, lymphocyte, and NLR), and cytokines (IL-10 and TNF-α), with PaO_2_/FiO_2_. The significant negative correlations (*P* < 0.001) with PaO_2_/FiO_2_ were evident for all parameters, except lymphocyte. The strength of their associations was as follows: age (*r* = –0.269), BMI (*r* = –0.236), CRP (*r* = –0.699), neutrophil (*r* = –0.584), NLR (*r* = –0.582), IL-10 (*r* = –0.752), and TNF-α (*r* = –0.752). Conversely, a strong positive correlation was observed between the lymphocyte count and PaO_2_/FiO_2_ ratio (*r* = 0.524, *P* < 0.001). The details of the correlations between other variables were depicted in Table 2.

**Table 2:** Correlation between parameters and PaO_2_/FiO_2_.

Parameters	*r* and *P*-values	PaO_2_/FiO_2_
IL-10	*r*	–0.752**
	*P*-value	<0.001
BMI	*r*	–0.236**
	*P*-value	<0.001
CRP	*r*	–0.699**
	*P*-value	<0.001
Age	*r*	–0.269**
	*P*-value	<0.001
TNF-α	*r*	–0.504**
	*P*-value	<0.001
Lymphocyte	*r*	0.524**
	*P*-value	<0.0018
Neutrophil	*r*	–0.584**
	*P*-value	<0.001
NLR	*r*	–0.582**
	*P*-value	<0.001

The correlation was done via a Pearson correlation test. The degree of correlation was done via correlation coefficient (*r*). A statistical significance was assumed if the *P*-value was less than 0.05. A single asterisk (*) denoted a situation where the *P*-value was less than 0.05; a *P*-value below 0.01 was symbolized by two asterisks (**); three asterisks (***) represented a *P*-value less than 0.001; and four asterisks (****) signified a *P*-value smaller than 0.0001. r: correlation coefficient; BMI: body mass index; CRP: C-reactive protein; IL-10: interleukin-10; TNF-α: tumor necrosis factor-alpha; NLR: neutrophil to lymphocyte ratio; PaO_2_/FiO_2_: partial pressure of oxygen/fraction of inspired oxygen.

### Utilizing multiple linear regression to analyze independent associations between the depression of PaO_2_/FiO_2_ and the severity of ARDS in COVID-19 patients

**Figure 2:**
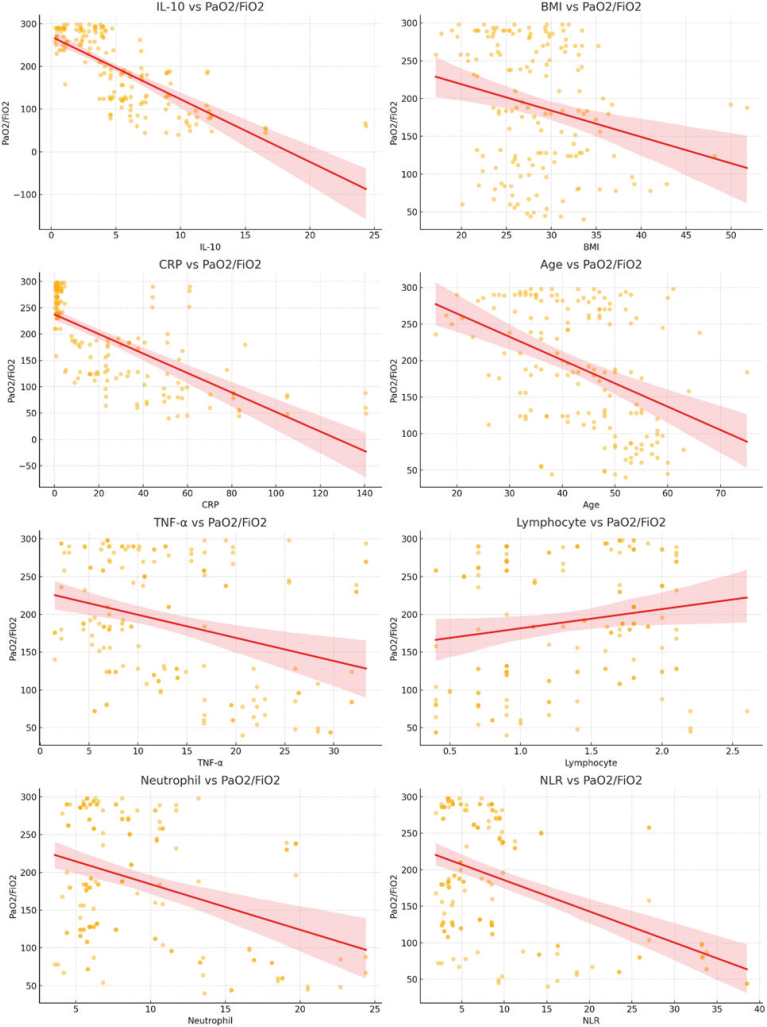
Scatter plot illustrating the relationship between the PaO_2_/FiO_2_ ratio and the severity of ARDS in COVID-19 patients. The PaO_2_/FiO_2_ ratio, an indicator of respiratory function and ARDS severity, is plotted as the dependent variable. Predictive factors considered include IL-10, BMI, CRP, age, TNF-α, lymphocyte count, neutrophil count, and the neutrophil-to-lymphocyte ratio (NLR). Each point represents an individual patient, with corresponding levels of independent variables. The distribution reflects how these clinical and biochemical markers collectively influence oxygenation impairment in COVID-19-induced ARDS. Statistical analysis highlights significant associations, emphasizing the role of inflammation and patient-specific factors in predicting ARDS severity. The scatter plot was generated using Python (Matplotlib). IL-10: interleukin-10; CRP: C-reactive protein; TNF-α: tumor necrosis factor-alpha; NLR: neutrophil to lymphocyte ratio; ARDS: Acute respiratory distress syndrome; BMI: body mass index; PaO_2_/FiO_2_: partial pressure of oxygen/fraction of inspired oxygen.

**Figure 3:**
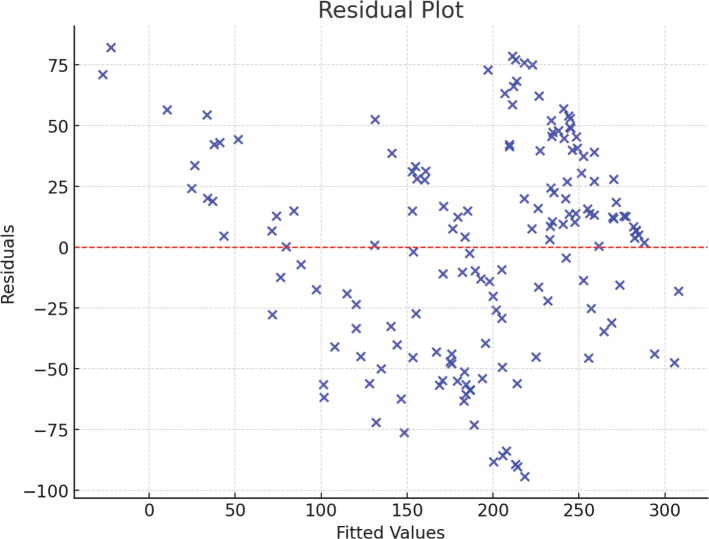
Residual Plot for the Multiple Linear Regression Model Predicting PaO_2_/FiO_2_. This residual plot illustrates the relationship between the fitted values (predicted PaO_2_/FiO_2_) and the residuals (differences between observed and predicted values) in the regression model. The model uses IL-10, BMI, CRP, age, TNF-α, lymphocyte count, neutrophil count, and NLR as independent variables to predict PaO_2_/FiO_2_, a measure of respiratory function in ARDS patients with COVID-19. The horizontal red dashed line at zero indicates no residual error. Points scattered randomly around this line suggest a well-fitted model, while patterns or clustering could indicate potential issues such as non-linearity, heteroscedasticity, or omitted variables. This figure was created using Python, with the Statsmodels library for regression modeling and Matplotlib for visualization. IL-10: interleukin-10; CRP: C-reactive protein; TNF-α: tumor necrosis factor-alpha; NLR: neutrophil to lymphocyte ratio; ARDS: Acute respiratory distress syndrome; BMI: body mass index; PaO_2_/FiO_2_: partial pressure of oxygen/fraction of inspired oxygen.

A statistical analysis using multiple linear regression was performed on a group of 158 patients to evaluate the prognostic significance of laboratory indicators, like the reduction in the PaO_2_/FiO_2_ ratio and the intensity of ARDS, for COVID-19 patients who are suffering from ARDS. In order to check the calibration of the model, an analysis was performed to determine the presence of multicollinearity in the relevant variables through the assessment of tolerance and variance inflation factor (VIF). Regarding linearity, we confirmed that the assumptions for linear regression were met, including the linear relationship between the dependent and independent variables. Linearity was tested using scatterplots of residuals versus predicted values, which showed no significant deviation from linearity (Figures 2 and 3). The depression of PaO_2_/FiO_2_, serving as the dependent variable, was analyzed using IL-10, BMI, CRP, age, TNF-α, lymphocyte, neutrophil, and NLR as independent variables.

The association between PaO_2_/FiO_2_ as the dependent variable and the independent variables (IL-10, BMI, CRP, age, TNF-α, lymphocyte, neutrophil, and NLR) was assessed (Table 3). IL-10 exhibited the strongest negative association with PaO_2_/FiO_2_ (Standardized B = -0.487, *P* < 0.001), indicating that increased levels of this anti-inflammatory cytokine may contribute to the deterioration of oxygenation in COVID-19-related ARDS. BMI also showed a significant negative association (Standardized *B* = -0.137, *P* = 0.002), suggesting that obesity may play a role in worsening ARDS outcomes. CRP, a key marker of systemic inflammation, was significantly associated with a decrease in PaO_2_/FiO_2_ (Standardized B = -0.323, *P* < 0.001). Elevated CRP levels reflect a heightened inflammatory state, which may contribute to lung injury, alveolar damage, and reduced gas exchange efficiency in ARDS. NLR, another significant predictor (Standardized B = -0.261, *P* = 0.003), further supports the role of inflammation in the progression of ARDS. A high NLR indicates an imbalance in immune responses, characterized by increased neutrophil activity and lymphocyte depletion, both of which have been linked to poor respiratory outcomes in COVID-19 patients. Other variables, including age (*P* = 0.059), TNF-α (*P* = 0.076), lymphocyte count (*P* = 0.072), and neutrophil count (*P* = 0.187), were not statistically significant predictors of PaO_2_/FiO_2_ reduction.

**Table 3:** Utilizing multiple line regression to know the association of the depression of PaO_2_/FiO_2_ and the severity of ARDS in COVID-19 patients.

Dependent variable	Independent variables	Standardized B*	95.0% Confidence interval for standardized B		*P*-value
PaO_2_/FiO_2_	IL-10	–0.487	–0.586	–0.387	<0.001
	BMI	–0.137	–0.223	–0.051	0.002
	CRP	–0.323	–0.43	–0.217	<0.001
	Age	–0.089	–0.182	0.003	0.059
	TNF-α	–0.091	–0.192	0.01	0.076
	Lymphocyte	–0.125	–0.262	0.012	0.072
	Neutrophil	0.099	–0.048	0.245	0.187
	NLR	–0.261	–0.435	–0.087	0.003

The regression analysis was conducted using SPSS 29. The depression of PaO_2_/FiO_2_, acting as the dependent variable, and the severity of ARDS in COVID-19 patients were associated with using IL-10, BMI, CRP, age, TNF-α, lymphocyte, neutrophil, and NLR as independent variables. A statistical significance was assumed if the *P*-value was less than 0.05. B: regression coefficient; BMI: body mass index; CRP: C-reactive protein; IL-10: interleukin-10; TNF-α: tumor necrosis factor-alpha; NLR: neutrophil to lymphocyte ratio; PaO_2_/FiO_2_: partial pressure of oxygen/fraction of inspired oxygen; ARDS: Acute respiratory distress syndrome.

* Change in PaO_2_/FiO_2_ per one standard deviation increase of biomarker.

### Utilizing ROC curve in analyzing associations to the severity of ARDS in COVID-19 patients

**Figure 4:**
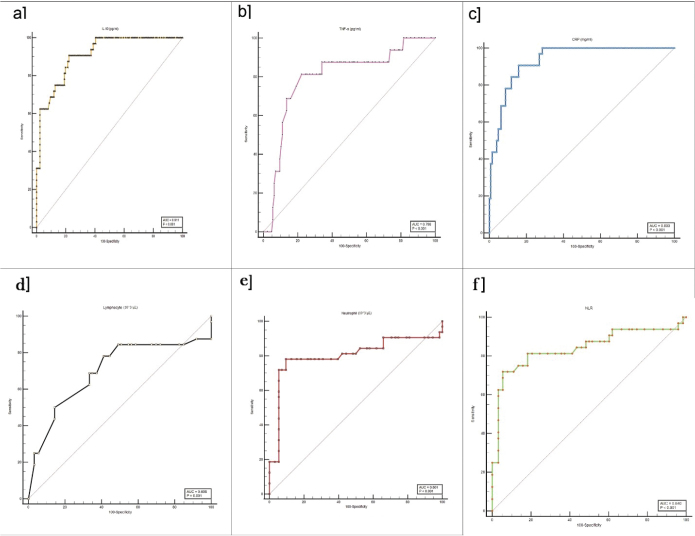
Assessing the ROC curve for laboratory biomarkers (a) IL-10, (b) TNF-α, (c) CRP, (d) Lymphocyte, (e) Neutrophil, and (f) NLR in associating with the severity of ARDS in COVID-19 patients. The graph of the ROC curve was drawn using MedCalc 20. The COVID-19 cases were categorized into non-severe (mild-to-moderate) ARDS and severe ARDS. IL-10: interleukin-10; TNF-α: tumor necrosis factor-alpha; NLR: neutrophil to lymphocyte ratio; CRP: C-reactive protein; ARDS: Acute respiratory distress syndrome; ROC: receiver operating characteristic.

To evaluate the prognostic potential of certain blood test markers (CRP, IL-10, TNF-α, neutrophil, NLR, and lymphocyte count) in determining the severity of ARDS in COVID-19 patients, ROC curves were created using data from 158 individuals with COVD-19. Of these patients, 126 were categorized as having non-severe (ranging from mild to moderate) ARDS, while the remaining 32 were classified as having severe ARDS. All laboratory markers were significantly associated with the severity of ARDS in COVID-19. Nevertheless, IL-10 (AUC = 0.911) and CRP (AUC = 0.933) are the markers with the highest AUC values. The cut-off values for IL-10 and CRP are >6.07 and >33.68, respectively. The sensitivity and specificity values of IL-10 are 90.62 and 77.78%, respectively. Additionally, CRP demonstrates a sensitivity of 90.62% and a specificity of 84.13%. The details of the ROC curve for different lab indicators are presented in Table 4 and illustrated in Figure 4.

**Table 4:** Evaluation of the ROC curve for laboratory biomarkers in predicting the severity of ARDS among individuals with COVID-19.

Parameters	Cut-off	AUC	95% CI	*P*-value	Sensitivity	Specificity	PPV	NPV
IL-10	>6.07	0.911	0.856–0.951	<0.001	90.62	77.78	50.9	97.0
TNF-α	>15.43	0.798	0.726–0.857	<0.001	81.25	77.78	56.4	91.6
CRP	>33.68	0.933	0.882–0.966	<0.001	90.62	84.13	59.2	97.2
Lymphocyte	≤1.2	0.698	0.620–0.769	0.001	78.12	58.73	32.5	91.4
Neutrophil	>10.8	0.801	0.731–0.861	<0.001	78.12	90.48	67.6	94.2
NLR	>11.24	0.840	0.774–0.894	<0.001	71.87	94.44	76.7	93.0

The analysis of the ROC curve was conducted using MedCalc 20. The COVID-19 cases were categorized into nonsevere (mild-to-moderate) ARDS and severe ARDS. A statistical significance was assumed if the *P*-value was less than 0.05. AUC: area under curve; CI: confidence interval; IL-10: interleukin-10; CRP: C-reactive protein; TNF-α: tumor necrosis factor-alpha; ROC: receiver operating characteristic; PPV: positive predictive value; NPV: negative predictive value; NLR: neutrophil to lymphocyte ratio; ARDS: Acute respiratory distress syndrome.

Both IL-10 and CRP showed high sensitivity, reaching 90.62%. CRP demonstrated greater specificity (84.13%) than IL-10 (77.78%). The PPV for IL-10 was 50.9%, and for CRP, it was 59.2%. IL-10 and CRP each showed high NPVs 97.0 and 97.2%, respectively.

### Associations of –1,082 A/G SNP IL-10 to the severity of ARDS in COVID-19 patients

Among severe ARDS cases (*n* = 32), the AA genotype was observed in 34.38% of patients, while the AG genotype was found in 62.50%, and the GG genotype in only 3.13%. In contrast, non-severe ARDS cases (*n* = 126) showed a higher proportion of the AG genotype (65.08%) and the GG genotype (22.22%), with only 12.70% carrying the AA genotype.

The association between IL-10 –1,082 A/G polymorphism and the severity of ARDS in COVID-19 patients was evaluated using both unadjusted (chi-square) and adjusted (logistic regression) analyses (Table 5). In the unadjusted model, the AG genotype showed a significantly lower odds of severe ARDS compared to the AA genotype (OR = 0.355, 95% CI: 0.151–0.889, *P* = 0.05). After adjusting for age, gender, and BMI, the protective effect became stronge r and statistically more robust (aOR = 0.198, 95% CI: 0.066–0.600, *P* = 0.004). Similarly, the GG genotype exhibited a very strong protective effect in the unadjusted analysis (OR = 0.052, 95% CI: 0.005–0.380, *P* = 0.004); however, this association was attenuated and became marginally non-significant in the adjusted model (aOR = 0.130, 95% CI: 0.016–1.049, *P* = 0.055). The dominant genetic model (AG+GG vs AA) consistently demonstrated a significant association in both unadjusted (OR = 0.278, *P* = 0.001) and adjusted analyses (aOR = 0.231, *P* = 0.001), reinforcing the protective role of the G allele. Notably, the recessive model (GG vs AA+AG) showed a conflicting result: a significantly increased risk in the unadjusted model (OR = 8.857, *P* = 0.036) that reversed direction after adjustment (aOR = 0.130), aligning with the individual GG genotype results. This suggests that the unadjusted association may have been confounded by age or other covariates. At the allele level, carriers of the G allele had reduced odds of severe ARDS in both unadjusted (OR = 0.433, *P* = 0.003) and adjusted models (aOR = 0.231, *P* = 0.001). Overall, these findings support a protective association between the IL-10 –1,082 G allele and the severity of ARDS in COVID-19 and underscore the value of adjusted logistic regression analyses in accurately identifying genotype–phenotype relationships.

**Table 5:** Analysis of the association between IL-10 -1082 A/G polymorphism and the severity of ARDS in COVID-19, considering both adjusted and unadjusted effects.

Model	OR (Unadj)	95% CI (Unadj)	*P*-value (Unadj)	aOR (Adj)	95% CI (Adj)	*P*-value (Adj)
AG vs AA	0.355	0.151–0.889	0.05	0.198	0.066–0.6	0.004
GG vs AA	0.052	0.005–0.380	0.004	0.13	0.016–1.049	0.055
Dominant (AG+GG vs AA)	0.278	0.12–0.61	0.001	0.231	0.094–0.57	0.001
Recessive (GG vs AA+AG)	8.857	1.1–72.3	0.036	0.13	0.016–1.049	0.055
Allele G vs A	0.433	0.250–0.766	0.003	0.231	0.094–0.57	0.001

*OR*: Odds Ratio; aOR: Adjusted Odds Ratio; CI: Confidence Interval; IL-10: interleukin-10; ARDS: Acute respiratory distress syndrome; BMI: body mass index. Unadjusted ORs were calculated using chi-square tests. Adjusted ORs were derived from multivariable logistic regression models controlling for age, gender, and BMI. Genotype AA was used as the reference category. Dominant model compares AG+GG vs AA; the recessive model compares GG vs AA+AG; the allele model compares G vs A allele count. Statistical significance was considered at *P* < 0.05.

## Discussion

This study highlights key factors associated with ARDS severity in COVID-19 patients by integrating laboratory biomarkers and genetic analysis. Elevated CRP and NLR, coupled with increased TNF-α and IL-10 levels, were associated with severe ARDS. Reduced lymphocyte counts and PaO_2_/FiO_2_ ratios further distinguished moderate and severe cases. IL-10 and CRP emerged as the most robust biomarkers, demonstrating high association toward ARDS severity. COVID-19 patients with the IL-10 –1,082 A/G SNP, especially those with GG and AG genotypes and G allele, experienced less severe ARDS.

ARDS not only is typically associated with sepsis caused by bacterial infections but can also stem from viral infections like SARS-CoV-2. The development of ARDS in the context of SARS-CoV-2 is thought to occur through either the virus’s direct invasions on respiratory epithelial cells or through an amplified immune response (3) resulting in the overproduction of cytokines such as IL-10 and TNF-α as well as increased levels of neutrophils, CRP, and NLR, while lymphocyte count decreases (4–7). Hence, the assessment of laboratory biomarkers in COVID-19 patients with ARDS is important when predicting disease severity, facilitating early intervention, and preventing disease progression.

SARS-CoV-2 engages with angiotensin-converting enzyme 2 (ACE2) receptors on respiratory epithelial cells or immune cells, triggering the release of Damage-associated molecular pattern (DAMP) and prompting the secretion of inflammatory cytokines. The interplay between immune and epithelial cells can manifest in diverse clinical outcomes, including ARDS, disseminated intravascular coagulation, pneumonia, and the hyper-inflammatory condition known as cytokine storm (31). Patients first exhibit symptoms resembling the flu, which quickly progress to ARDS. ARDS involves abrupt and widespread inflammation within the alveolar-capillary barrier, leading to enhanced vascular leakage, diminished compliance, impaired gas exchange, and resultant hypoxemia (32, 33). One clinical parameter is the reduced PaO_2_/FiO_2_ ratio to a value below 300 mm Hg (34).

Our hypothesis is that ARDS from different viruses might present with different features. Ding and Chen’s 2022 study found that COVID-19 patients with ARDS had higher coagulation levels (like prothrombin time, fibrinogen, and D-dimer) than those with H7N9-ARDS. Septic shock and multiple organ dysfunction caused death more often in COVID-19-ARDS patients than in H7N9-ARDS patients (35). Bacterial sepsis and COVID-19 induce ARDS with distinguishable clinical and immunological characteristics. ARDS caused by bacterial sepsis is distinguished by its rapid onset, increased inflammatory markers (e.g. IL-6 and Procalcitonin [PCT]), and reduced systemic blood flow, often resulting in a more severe cytokine storm (36). In contrast, ARDS caused by COVID-19 is characterized by prolonged respiratory failure, moderate cytokine elevation, and notable damage to the endothelial cells leading to blood clot formation (36). Metabolic pathway differences, particularly in sphingosine and arginine metabolism, are highlighted by molecular analyzes, suggesting unique underlying mechanisms (37). These differences demand customized treatments; corticosteroids and anticoagulants work differently depending on what caused the ARDS (38).

In regard to hematological parameters and inflammation markers (CRP), our findings indicate that the neutrophil count, NLR, and CRP are elevated, while the lymphocyte count is reduced in the COVID-19 ARDS groups compared to the HCs. The ROC analysis demonstrated that neutrophil count, NLR, and CRP potentially can be used when monitoring the severity of ARDS in COVID-19. Furthermore, the analysis of multiple linear regression showed that NLR and CRP are independently associated with the depression of PaO_2_/FiO_2_. Our findings support the research conducted by Bano Mehdi et al., which demonstrated that NLR is indicative of the severity of ARDS in COVID-19 (6). The potential contribution of NLR to ARDS may be attributed to two mechanisms. First, when NLR is high, it indicates an increased number of neutrophils, which, in turn, release inflammatory cytokines, including IL-6 and TNF-α. These cytokines contribute to systemic inflammation and can exacerbate lung damage, particularly in conditions such as ARDS (39). Another important point to consider is that our findings corroborate the idea that NLR-induced systemic inflammation hinders the function of lymphocytes, which are key regulators in the immune response against ARDS. This ultimately leads to a more severe progression of COVID-19 (40). In relation to CRP, our results are consistent with the study conducted by Bano Mehdi et al., which revealed higher CRP levels in COVID-19 ARDS patients compared to those without ARDS (6).

In the current study, patients suffering from ARDS triggered by SARS-CoV-2 demonstrated elevated levels of TNF-α and IL-10 compared to those in the HC group. This result is consistent with the results reported by Smail et al., revealing elevated cytokine levels in the severe and critical COVID-19 groups as opposed to the mild and HC groups (41). Furthermore, IL-10 has the potential to predict the depression of PaO_2_/FiO_2_ and the severity of ARDS in COVID-19. In addition to its classical anti-inflammatory effects, there are two proposed mechanisms for the increase of IL-10 in ARDS COVID-19 patients. First, IL-10, released by regulatory T-lymphocytes (Treg), is upregulated as a negative feedback mechanism in an attempt to attenuate the hyper-inflammatory response observed in ARDS. However, it is unable to fully control the inflammation. Furthermore, our previous research paper supports the notion that IL-10, produced by monocytes and macrophages, exerts a proinflammatory effect in COVID-19 (41).

Patients with Crohn’s disease, who were administered high doses of IL-10, experienced increased inflammation due to elevated levels of Interferon-gamma (IFN-γ) (42). Furthermore, it has been observed that the simultaneous administration of lipopolysaccharides (LPS) and IL-10 results in an elevation of inflammation by means of increased IFN-γ (43). It has been previously mentioned that secondary bacterial infections are commonly observed in severe cases of COVID-19, particularly with gram-negative bacteria containing LPS (44). In cases of ARDS associated with COVID-19, the co-occurrence of high levels of IL-10 and LPS can lead to a state of hyper-inflammation. Furthermore, IL-10 triggers inflammation by stimulating B-lymphocytes to produce auto-antibodies. In the absence of IL-2, IL-10 promotes the growth of T-lymphocytes and inhibits T-lymphocyte apoptosis (45).

This study suggests that both AG and GG genotypes and G allele contribute to the decrease of ARDS severity. This finding is not in line with the results of Abbood, Anvari (46), who found that AG and GG genotypes increase COVID-19 mortality. This observation also contradicts the outcome reported by Rizvi, Rizvi (47), who found no link between the severity of COVID-19 and the AG genotype, GG genotype, or G allele. The present findings also do not correspond with the results of Gong, Taylor (48). They identified GG as a risk factor for ARDS development in elderly hospitalized patients. However, in the context of ARDS severity, Gong, Taylor (48) in line with our study reported that the GG genotype was protective and associated with decreased in ARDS severity in non-COVID-19 patients. Variations in study design, sample sizes, patient demographics, disease stages, and treatment protocols could be part of the explanation of these diverging results.

In the present study, IL-10 and CRP both demonstrated high sensitivity (90.62%), indicating these markers effectively detect severe ARDS cases. CRP displayed a higher specificity (84.13%) compared to IL-10 (77.78%), suggesting CRP is more reliable in ruling out non-severe cases. IL-10 showed a PPV of 50.9%, while CRP demonstrated 59.2%. This indicates that CRP is slightly better at predicting true severe cases when the test is positive. The relatively moderate PPVs may reflect the overall lower prevalence of severe ARDS in the cohort, which can reduce PPV despite high sensitivity and specificity. Both IL-10 and CRP displayed high NPVs (97.0 and 97.2%, respectively), meaning that a negative result strongly predicts the absence of severe ARDS.

This study is subject to certain limitations. First, the sample size is relatively small. The limited sample size means that there is a risk that true differences were not detected. Furthermore, this study does not include a COVID-19 group without ARDS and an ARDS group without SARS-CoV-2 infection, which limits the ability to identify biomarkers associated with ARDS development. Another limitation is that the variant of SARS-CoV-2 remained unidentified. The absence of severity scores for ICU patients, such as Acute Physiology and Chronic Health Evaluation II (APACHE II), Simplified Acute Physiology Score (SAPS), Sequential Organ Failure Assessment (SOFA), Mortality Probability Model (MPM), Multiple Organ Dysfunction Syndrome (MODS), and Pulmonary Severity Scoring System (PSSS), also poses a potential opportunity for improvement. Finally, this study potentially lacks external validity because it does not compare genetic profiles to other populations inside or outside Iraq. This study may have residual confounding, as it might not consider all possible variables like socio-economic status or healthcare access.

Despite previous constraints, in this study, the only one of its kind in Iraq robustly assesses COVID-19 ARDS severity using genetic markers (IL-10 SNP). These findings could significantly improve clinical practice. By leveraging demographic factors, hematological parameters, inflammatory biomarkers, cytokines, and the –1,082 A/G polymorphism, healthcare practitioners can optimize risk stratification, enhance prognostic accuracy, and tailor therapeutic approaches for patients with COVID-19-associated ARDS, ultimately improving clinical outcomes and mitigating disease burden.

## Conclusion

Our findings highlight the critical role of various laboratory biomarkers, including hematological indices such as Neutrophil count and NLR as well as inflammation biomarkers like CRP and serum IL-10, in monitoring the severity of ARDS in COVID-19 patients. Moreover, the identification of the –1,082 A/G SNP in the IL-10 gene as a potential protective genetic determinant against ARDS severity in COVID-19 underscores the importance of personalized medicine approaches. This study emphasizes the role of genetic variation in IL-10, particularly the protective effect of the GG and AG genotypes and G allele, and highlights age as a key determinant in the clinical progression of ARDS among COVID-19 patients.

## Ethics approval and consent to participate

Research protocols were accepted by the ethical committee (No. 01062021-5-9; approved on May 5, 2021) at the University of Duhok and Ministry of Health/Directorate General of Health-Duhok. It is consistent with the Declaration of Helsinki. Each participant was presented with a consent form, and if the patients were unable to sign due to the severity of their illness or disability, their legal representatives were asked to affix their signature.

## Supplementary Material



## Data Availability

The corresponding author can provide the datasets used and/or analyzed during the current study upon reasonable request.
