# Supplementary material for: ARDS severity in COVID-19: a case–control study of laboratory biomarkers and IL-10 SNP analysis
Source: Ups J Med Sci. 2025 Jul 7;130:10.48101/ujms.v130.11515. doi: 10.48101/ujms.v130.11515 (PMC12320925; doi:10.48101/ujms.v130.11515)
Supplement: Supplementary file 1 [file UJMS-130-11515-s1.pdf]

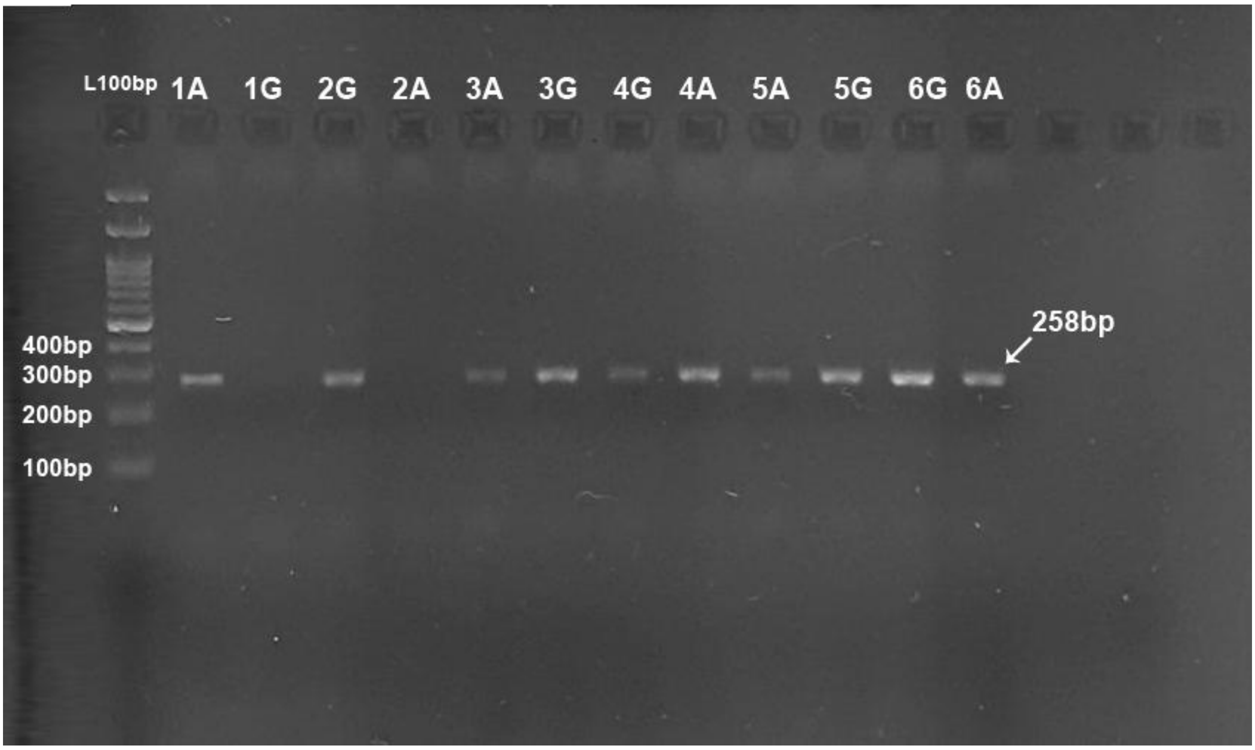

Supplementary figure 1. Gel electrophoresis result of-1082 A/G SNP in the IL-10 PCR products. The marker used had a length of 100 base pairs. Each sample underwent testing using two reaction tubes. Tubes A1 and G1 were utilized for the first sample, tubes A2 and G2 for the second sample, and this pattern continued up to the sixth sample. If the band produced in both tubes is 258 bp, it suggests that the sample has an AG genotype, and samples 3 to 6 also have an AG genotype. Additionally, if the 258 band is only observed in the G allele reaction tube, it suggests a GG genotype, as seen in the sample 2. On the other hand, if only the A allele reaction tube shows a band at 258, it suggests an AA genotype, like sample 1. L100bp: ladder 100 bp, A: allele A, G: allele G.

Supplementary Table 1. The primers employed to determine the -1082A/G SNP of IL-10 in this study (26, 27).

Primers	Primer sequences 5′-3′	Band size
common Forward primer	CAG TGCCAA CTG AGAATT TGG	-
reverse primer specific (G allele)	CTACTA AGG CTTCTTTGG GAG	258bp
reverse primer specific (A allele)	CAG TGCCAACTG AGAATT TGG	258bp

Supplementary Table 2: Multiple Comparisons between HCs and ARDS COVID-19 Groups

Dependent Variable	Groups (I)	Groups (J)	Mean Difference (I-J)	SEM	p-value	95% Confidence Interval
IL-10	HCs	Mild ARDS	-0.91	0.37	0.066	-1.87 to 0.04
	HCs	Moderate ARDS	-5.12	0.40	<0.001***	-6.17 to -4.08
	HCs	Severe ARDS	-9.30	0.48	<0.001***	-10.53 to -8.06
BMI	HCs	Mild ARDS	0.19	0.82	0.995	-1.94 to 2.32
	HCs	Moderate ARDS	-3.63	0.90	<0.001***	-5.96 to -1.29
	HCs	Severe ARDS	-2.73	1.06	0.053	-5.50 to 0.02
CRP	HCs	Mild ARDS	-4.83	4.84	0.750	-17.37 to 7.69
	HCs	Moderate ARDS	-66.12	5.30	<0.001***	-79.84 to -52.38
	HCs	Severe ARDS	-67.74	6.27	<0.001***	-83.97 to -51.50
TNF-α	HCs	Mild ARDS	-6.20	1.02	<0.001***	-8.84 to -3.56
	HCs	Moderate ARDS	-3.54	1.11	0.009	-6.43 to -0.65
	HCs	Severe ARDS	-14.05	1.32	<0.001***	-17.47 to -10.63
NLR	HCs	Mild ARDS	-5.48	0.84	<0.001***	-7.66 to -3.30
	HCs	Moderate ARDS	-3.25	0.92	0.003	-5.64 to -0.86
	HCs	Severe ARDS	-16.87	1.09	<0.001***	-19.70 to -14.04

Post-hoc Tukey HSD test results (statistical significance: $p < 0.05$). Three asterisks (***) in the table indicate statistical differences between the groups, as specified under the p-value column in Table 1.

BMI: Body Mass Index, CRP: C-reactive protein, HC: Healthy Controls, IL-10: Interleukin-10, TNF-α: Tumor Necrosis Factor-alpha, NLR: Neutrophil to Lymphocyte Ratio, ARDS: Acute Respiratory Distress Syndrome, SEM: Standard Error of Mean.
